# Online Nodal Demand Estimation in Branched Water Distribution Systems Using an Array of Extended Kalman Filters

**DOI:** 10.3390/s25082632

**Published:** 2025-04-21

**Authors:** Francisco-Ronay López-Estrada, Leonardo Gómez-Coronel, Lizeth Torres, Guillermo Valencia-Palomo, Ildeberto Santos-Ruiz, Arlette Cano

**Affiliations:** 1 Tecnológico Nacional de México, IT Tuxtla Gutiérrez, Turix-Dynamics Diagnosis and Control Group, Carr. Panam. S/N, Tuxtla Gutiérrez 29050, Mexico; frlopez@tuxtla.tecnm.mx (F.-R.L.-E.); leonardo.gc@tuxtla.tecnm.mx (L.G.-C.);; 2 Instituto de Ingeniería, Universidad Nacional Autónoma de México, Ciudad de México 04510, Mexico; ftorreso@iingen.unam.mx; 3 Tecnológico Nacional de México, IT Hermosillo, Av. Tec. 115, Hermosillo 83170, Mexico

**Keywords:** water demand, branched water distribution system, pipeline monitoring, unknown-input observer, extended Kalman filter

## Abstract

This paper proposes a model-based methodology to estimate multiple nodal demands by using only pressure and flow rate measurements, which should be recorded at the inlet of the distribution system. The algorithm is based on an array of multiple extended Kalman filters (EKFs) in a cascade configuration. Each EKF functions as an unknown input observer and focuses on a section of the pipeline. The pipeline model used to design the filters is an adaptation of the well-known rigid water column model. Simulation and experimental tests on standardized pipeline systems are presented to demonstrate the proposed method’s effectiveness. Finally, for the case of the experimental validation, both steady-state and variable input conditions were considered.

## 1. Introduction

The supply of water faces different problems, ranging from the overexploitation of aquifers, problems in water distribution networks, and pollution, to the lack of control over concessions. These problems affect different sectors of the population and economic activities such as construction or agriculture. In recent years, intensive research has been carried out to improve the monitoring and management of water distribution systems (WDSs) by estimating various parameters that help in the calibration of models with high spatial and temporal resolution [1,2]. Accurate knowledge of nodal demands that vary at different times is a prerequisite for predicting a distribution system’s pressure and quality. Consequently, determining these demands helps to improve a distribution system’s performance for the network operators [3]. Pressures and flows in distribution networks vary according to the nodal demands, which are generally not measurable. Therefore, their estimation is a problem of vital importance in managing WDSs.

Estimation of these demands is carried out from measured states, and it can be seen as an inverse problem whose solution depends on how many state variables are measurable. In this context, in the work performed by [4], a digital-twin-based solution was proposed to estimate the unsteady flow states in a pumping station. In this approach, the digital twin utilizes a combination of frequency domain analysis and predictive control theory to accurately estimate real-time hydraulic parameters at locations within the pumping station where sensors are not available, even in noisy and uncertain conditions.

Ideally and technologically, it is possible to place sensors in an entire hydraulic network; however, system costs make implementing complete hydraulic instrumentation prohibitive. This problem has been addressed in the literature using optimal sensor placement [5,6] or more recently, in work performed in [7], where a hybrid approach to sensor placement and state estimation applied to water distribution networks was developed, obtaining a mean absolute percentage error of less than 5% in the estimation of the pressure head available in the nodes of the WDS. However, even though this technique significantly reduces instrumentation costs, it does not provide information about the demand at non-instrumented nodes. Therefore, the online estimation of nodal demands is an open problem.

One way to tackle this problem is through vibration signals [8,9], acoustic signals [10], and virtual sensors, such as unknown input observers or extended Kalman filters (EKFs), among others. In particular, the Kalman filter is a commonly used mathematical tool for real-time state estimation [11]. In this work, a virtual sensor approach is considered. These algorithms allow estimating unknown states, i.e., the demands, based on the measurement of certain available inputs and outputs [12]. Some reported results can be found in the literature; for instance, in [3], the authors estimated node demands using particle swarm optimization. The authors in [13] proposed the Davidon–Fletcher–Powell algorithm, which considers a one-dimensional optimization and scale matrix calculation. Other authors have proposed methodologies based on an extended Kalman filter.

In [14], an extended Kalman filter approach for adaptative calibration of the nodal demands in water distribution systems was introduced. Twenty-four-hour demand patterns were introduced as prior information for the prediction module and then measurement data were used in the correction module to determine the final estimation of the nodal demand, demonstrating an improved calibration accuracy and robustness against measurement noise. In [15], a two-objective sampling design method was used for the improvement of the observability of the system and to mitigate the effect of noise in sensor measurements. This was used in the context of nodal demand estimation, since proper sensor sampling is essential for the estimation of nodal demands in a water distribution network, as well as for improving the model accuracy. The work performed in [16] focused on the estimation of short-term demands in a water distribution network by means of AI techniques, to support sustainable water management. The study reported a comparison between nine machine learning and deep learning methods, demonstrating that Long-Short Term Memory (LSTM) neural networks provided an increased accuracy, even in real-time predictive applications.

In [17], as in [18], the authors suggested the design of EKFs based on a hydraulic model to estimate a leak’s magnitude and location. In [19], a comparison of different short-term water demand forecasting models based on patterns, probability, and moving window techniques was proposed. The EKF stood out from other observers due to its functionality in predicting unknown states under measurement noise and model mismatches, as can be consulted in the survey [20] and references therein. The authors in [21] proposed a method based on a dual model that automatically converts slight pressure deviations caused by leaks into signals in the form of virtual leaks. A more in-depth revision of the method for leak localization can be consulted in the recent survey in [22] and references therein. However, although leakage estimation involves flow estimation, few works have focused on nodal demand estimation.

For instance, in [23], a calibration method for demands based on an optimization methodology was proposed. In [24], a probabilistic method for node demand evaluation was analyzed; as a result, the transient response and wave propagation in a pipeline were determined. Based on previous works, the authors in [25] proposed a transient-based method to skeletonize pipes in series with internal demands. A methodology for node pressure estimations based on artificial neuronal networks was proposed in [26]. Based on EPANET models and historical data, an optimization-based approach for water consumption was developed in [27]. Recently, a stochastic approach for analyzing demand in water distribution systems was proposed in [28]. It is important to note that, despite the different approaches reported in the literature, most works have been based on offline estimations or EPANET models, reducing their applicability.

This work proposes an estimation technique for the demands of a WDS based on extended Kalman filters. The proposed method considers a nonlinear model and the dynamic behavior of the WDS. The method was proved experimentally on a pilot hydraulic network at the Hydroinformatics Laboratory at the Institute of Technology of Tuxtla Gutiérrez. The system’s instrumentation allowed us to validate the algorithms experimentally. Additionally, it was demonstrated that the proposed algorithm presented robustness to measurement noise, which enhances its applicability.

In the literature, approaches such as particle swarm optimization (PSO) and probabilistic estimation have been successfully applied to similar problems. Nonetheless, they often include high computational complexity and become difficult to adapt to real-time scenarios with rapidly changing dynamics. On the other hand, EKFs are a computationally efficient framework for recursively estimating the states of nonlinear systems with complex dynamics and Gaussian noise, which is suitable for online applications where fast convergence and low-latency estimation are needed. Furthermore, EKFs can incorporate system models and online sensor measurements, enabling robust performance estimation, even under uncertain or partially observable conditions. These advantages make EKFs a robust and computationally efficient alternative for online estimation compared to other model-based approaches.

The remainder of this document is organized as follows: Section 2 presents some key facts about the nonlinear model. Section 3 presents the node demand algorithm based on the extended Kalman filter. Section 4 validates the method experimentally in the pilot plant. Finally, conclusions are given in Section 5.

## 2. Water Distribution Networks

**Figure 1:**
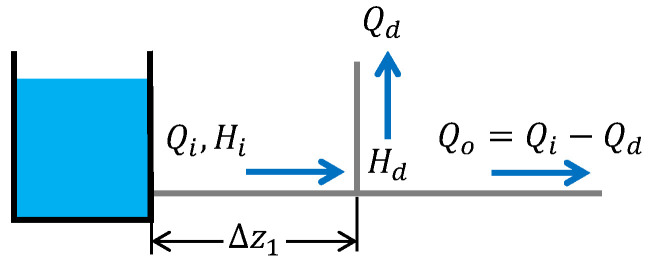
Schematic diagram of a unique demand in a branched pipeline.

A WDS aims to deliver water from reservoirs to the usage point with adequate quantity and pressure. It comprises interconnected pipes and can be classified as a grid, ring, radial, or dead-end (also called branched) system. While the first three types can be seen as a closed configuration, more suitable for cities and small districts, a branched system is commonly used for main distribution and irrigation systems [29,30]. This work is dedicated to branched water distribution systems (BWDSs). A branched system is composed of a main pipeline, with branches connected to the mainline (see Figure 1). The amount of liquid that flows in the sections is determined by knowing the water demand at each node. However, at best, these computations are done a priori, before constructing the WDS [28]. Determining the water consumption of nodes is challenging and usually requires a significant economic and time investment. Node demands are usually considered inputs to the WDS and are of great importance for the correct distribution of water throughout the network. However, due to the aforementioned problems, they are usually assumed to be unknown, due to a limited number of sensors. This work aims to estimate these demands online using only the information provided by two sensors at the pipeline’s inlet and outlet.

### 2.1. Modeling

**Figure 2:**
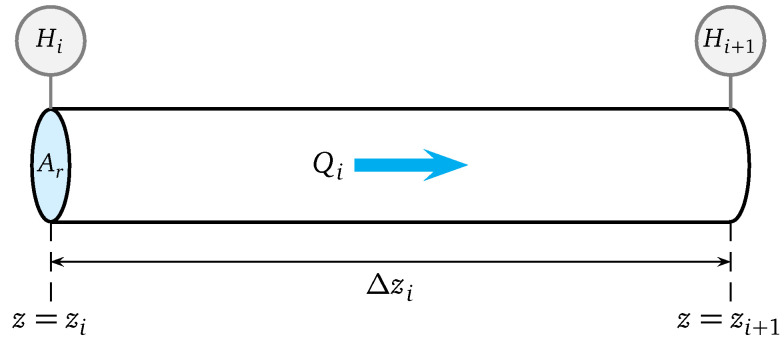
A simple pipeline scheme.

In order to design a node-demand estimator, a mathematical model based on the rigid water column (RWC) theory is considered; i.e., it is assumed that the entire fluid column moves as a rigid body, as illustrated in Figure 2.

The following assumptions are taken into account [31,32]: (i) the flow is assumed to be one-dimensional, (ii) the cross-sectional area is constant along the pipe, (iii) the pipe walls are rigid, (iv) the liquid fluid is incompressible, and (v) the convective changes in velocity are negligible. Then, under such assumptions, the RWC model that describes the flow dynamics in a pipeline section with size $\Delta z_{i}$ is given by

(1) $${\dot{Q}}_{i}=gA_{r}\frac{\Delta H_{i}}{\Delta z_{i}}-\alpha \left|Q_{i}\right|Q_{i}^{\gamma },$$

where $Q_{i}$ (m^3^/s) is the flow rate through the *i*-th pipe section, $\Delta H_{i}=H_{i}-H_{i+1}$ (m) is the head loss along section *i* with size $\Delta z_{i}$ (m), *g* (m/s^2^) is the gravitational acceleration, and $A_{r}$ (m^2^) is the cross-sectional area. α and γ≤1 are dimensionless parameters related to the friction losses, which are associated with the physical parameters of the pipe and the fluid.

By considering γ=1 and $\Delta H=H_{i}-H_{i+1}$, where $H_{i}$ and $H_{i+1}$ are the section inlet and outlet hydraulic heads, respectively, and assuming that the flow rate is one-directional, Equation (1) can be rewritten as [12]:

(2) $${\dot{Q}}_{i}=-\alpha Q_{i}^{2}+\frac{gA_{r}}{\Delta z_{i}}\left(H_{i}-H_{i+1}\right).$$

If a nodal demand is at the end of the *i*-th section, then the previous equation can be rewritten as

(3) $${\dot{Q}}_{i}=-\alpha Q_{i}^{2}+\frac{gA_{r}}{\Delta z_{i}}\left(H_{i}-H_{d}\right),$$

where $H_{d}$ is the hydraulic head at the demand node and is given by

(4) $$H_{d}={\left(\frac{Q_{d}}{r\left(\sigma \right)A_{d}}\right)}^{2}\left(\frac{1}{2g}\right),$$

where $Q_{d}$ is the node demand, $A_{d}$ is the node cross-sectional area, r(σ)∈[0,1]∈R is a coefficient that is capable of adjusting the consumption $Q_{d}$, much like one regulates the water flow by opening or closing the water taps associated with the node [33].

Equation (4) can be rewritten as

(5) $$H_{d}=\lambda \left(\sigma \right)\;Q_{i}^{2},$$

where $\lambda \left(\sigma \right)=\kappa^{2}/\left(2gr\left(\sigma \right)A_{d}\right)$ and κ∈(0,1) is a multiplier that proportionally relates $Q_{d}$ with $Q_{i}$.

By substituting (5) into (3), the following model, which represents a pipeline with one demand, is obtained:

(6) $${\dot{Q}}_{i}=-\alpha Q_{i}^{2}+\frac{gA_{r}}{\Delta z_{i}}H_{i}-\frac{gA_{r}}{\Delta z_{i}}\lambda \left(\sigma \right)Q_{i}^{2},$$

where λ(σ) can be seen as an unknown input of system (6) that needs to be estimated to calculate $Q_{d}$. By defining the state vector $x={\left[x_{1},\;x_{2}\right]}^{⊤}={\left[Q_{i},\;\lambda \left(\sigma \right)\right]}^{⊤}$, the following dynamic model is obtained:

(7) $$\begin{array}{cc} {\dot{x}}_{1} & =-\alpha \;x_{1}^{2}+\frac{gA_{r}}{\Delta z_{1}}H_{i}-\frac{gA_{r}}{\Delta z_{1}}x_{2}\;x_{1}^{2},\\ {\dot{x}}_{2} & =0,\\ y & =x_{1}. \end{array}$$

### 2.2. Demand Estimation

To estimate the demand at each node of the branched pipe, it is necessary to take into account the following points:

1. The sum of the flow rates between demand nodes is equal to the total demand measured at the inlet of the pipeline, i.e.,
  (8) $$Q_{in}=\sum_{j=1}^{N_{Q_{d}}}\kappa_{j}Q_{i}.$$
2. The demand at node *j* is proportionally related to the previous demand (i.e., to the demand at node j−1) in the following way:
  (9) $$Q_{d_{j}}=\kappa_{j}Q_{i}=\kappa_{j}\left(Q_{i-1}-Q_{d_{j-1}}\right).$$
3. λ(σ) is a function of two key parameters: κ and r(σ). This last parameter can take any real value between 0 and 1; if the node is fully open, it becomes 1, and if it is fully closed, it becomes 0. Therefore, these two values must be considered to design an algorithm for estimating the demands. As for κ, it is an unknown parameter that depends on the characteristics of the hydraulic installation after the node.

**Figure 3:**
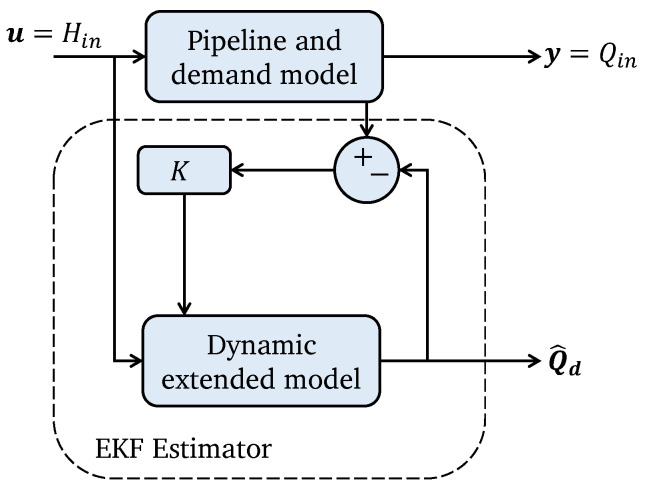
Extended Kalman filter strategy for estimation of an unknown demand.

To estimate the unknown state $x_{2}$ an extended Kalman filter (EKF) is considered, because it can minimize the estimation error by considering the measured inputs and outputs. The proposed methodology is illustrated in Figure 3.

To implement the EKF, we consider a discrete-time *process model* of the pipeline network:

(10) $$x_{k+1}=\Phi \left(x_{k},u_{k}\right)+w_{k},$$

where $x_{k}$ is the state vector, $u_{k}$ is the input vector, and $w_{k}$ is the *process noise* vector with zero mean and covariance *Q*, and with the *measurement model*:

(11) $$y_{k}=Cx_{k}+v_{k},$$

where $y_{k}$ is the measurement vector and $v_{k}$ is the *measurement noise* vector with zero mean and covariance *R*. Although the measurement model is a linear function, the process model Φ is a non-linear function of states and inputs. The covariance matrix *Q* is assigned empirically from the certainty or confidence in the process model Φ, i.e., the noise $w_{k}$ models uncertain inputs and/or unknown dynamics. The covariance matrix *R* is assigned based on the noise $v_{k}$ captured by the sensors, which is commonly associated with low resolution and data errors caused by various types of interference. To obtain *R*, the covariance of repeated steady-state sensor measurements is computed.

Based on a tradeoff between how much to trust the prediction (matrix *Q*) versus how much to trust the measurement (matrix *R*), the EKF aims to minimize the *estimation error covariance*; that is, it seeks to obtain the best possible estimate of the state of a dynamic system by minimizing the mean squared error (MSE). Mathematically, what the EKF minimizes is the trace of the error covariance matrix, tr(P), which is the sum of the variances of the errors across all state variables [34].

The EKF is implemented in two major steps for each time step: first, a *prior estimate* denoted by $x_{k+1}^{-}$ is obtained from the dynamic model; then, a *posterior estimate*$x_{k+1}$ is obtained by correcting the prior estimate using the measurements [35]. In the literature, the estimation of the forecast state $x_{k+1}^{-}$ is referred to as *prediction step*, and the update to obtain $x_{k+1}$ is referred to as the *data-assimilation step*, where measurement data are injected to improve the estimates. To begin the state estimation process, an initial estimate ${\hat{x}}_{0}^{-}$ and an error covariance matrix $P_{0}^{-}$ associated with the uncertainty of the initial estimate are provided. Over time, the state estimation error tends to reach a minimum covariance (i.e., a minimum uncertainty in the estimated state).

To apply the EKF iterative process, it is necessary to consider a discrete version of the system. In previous work, it has been demonstrated that the best discretization method, which provides the best trade-off between sampling and accuracy for a pipeline system, is Heun’s method [36]. Then, the discrete-time version of (7) is described by

(12) $$x_{k+1}=\underset{\Phi (x_{k},u_{k})}{\underset{︸}{x_{k}+\frac{T_{s}}{2}\left(\phi \left(x_{k},u_{k}\right)+\phi \left(x_{k}+T_{s}\;\phi \left(x_{k},u_{k}\right),u_{k}\right)\right)}},$$

where $T_{s}$ is the sample time, *k* is the discrete-time index, and ϕ is the continuous-time state-transition function given by

(13) $$\phi \left(x,u\right)={\left[-\alpha \;x_{1}^{2}-\frac{gA_{r}}{\Delta z}{x_{1}}^{2}\;x_{2}+\frac{gA_{r}}{\Delta z}u,\;0\right]}^{⊤}.$$

Additionally, considering that the noisy measurement of the first state variable ($Q_{in}$) is available at each time step, the matrix *C* in the observation model (11) is given by

(14) $$C=\left[\begin{array}{cc} 1 & 0 \end{array}\right]$$

From the discrete-time version given by the dynamic model in (12) and (13), and the observation model in (11) and (14), the unknown demand can be estimated as illustrated in Figure 3, which is also summarized in Algorithm 1 (Appendix A).
**Algorithm 1:** EKF algorithm for demand estimation
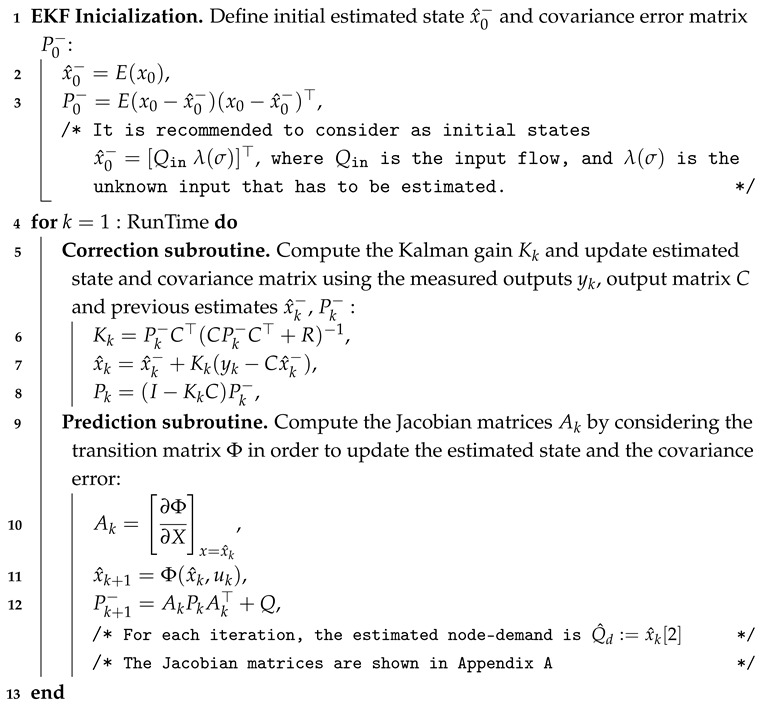


## 3. Extension to the Estimation of *n* Node-Demands

**Figure 4:**
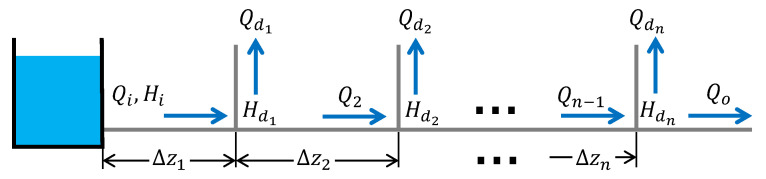
Branched distribution system with *n* demand nodes.

Now consider a system with *n* node demands, as illustrated in Figure 4. In this case, the problem is more complicated, assuming that it is only possible to have sensors in the inlet flow as in many real systems, which means that only $Q_{i}$ and $H_{i}$ are known.

Taking into account the same strategy as discussed above, if the node demands are estimated, then it is possible to estimate the flow after the demand as

(15) $$Q_{n}=Q_{n-1}-Q_{d_{n-1}},\;n=i,2,\ldots ,n.$$

**Figure 5:**
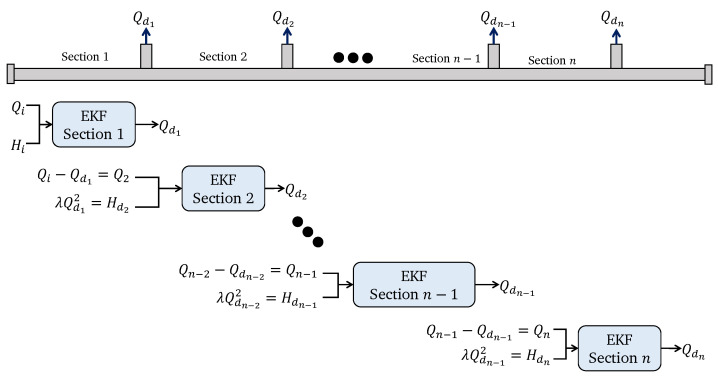
Cascade EKF strategy for the estimation of *n* node-demands.

Then, by sectioning the all-branched pipeline, where each section represents a node demand, it is possible to estimate all unknown node demands, as shown in Figure 5. This algorithm must be applied to each section of the pipe sequentially. Thus, after estimating the pressure of the demand node ($H_{d}$), this becomes the new input for the following section.

The reader should note that an EKF estimator must be computed for each section, as given in Algorithm 1. This design of sequential estimators can be used for monitoring WDSs, because it allows the online estimation of all the node demands. Moreover, the method uses the same mathematical model for all pipeline sections, which minimizes the computational cost. Furthermore, by considering the EKF, it is possible to achieve some robustness against model uncertainties and the inherent measurement noise.

## 4. Experimental Results

**Figure 6:**
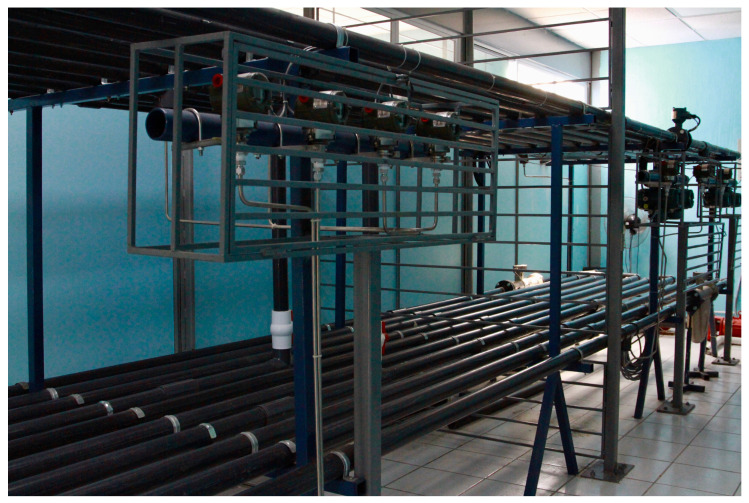
Experimental water distribution system.

This section considers a WDS with two branches located at the Hydraulic Laboratory at the Technological Institute of Tuxtla Gutierrez, as displayed in Figure 6.

The system consists of a reservoir with 2500 L capacity; 256 m of 2-inch PVC pipes; a hydraulic centrifugal pump with 5 hp power; a Siemens Micromaster 420 frequency converter for pump control; 2 ROTAMASS Total Insight Coriolis mass flow meters, placed at the inlet and outlet of the first level pipe, with 0.5% accuracy in data collection; 2 YOKOGAWA ADMAG AXR magnetic flow meters, located on the top floor of the network, with a resolution of 0.5%; 8 Yokogawa EJA530 industrial pressure transmitter sensors, strategically placed on both levels, with an accuracy of 0.55%; and a Yokogawa GM10 Dataloger connected to MATLAB. It is important to note that for the sake of this study, only a pressure sensor was considered for the experiments, and the rest were used for validation purposes only. The sampling rate of all measurement devices is 10 Hz, corresponding to 10 samples per second. The physical parameters of the system are summarized in Table 1.

**Figure 7:**
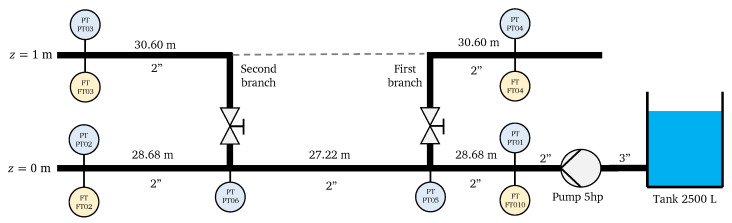
Piping and instrumentation diagram of the experimental WDS.

The piping and instrumentation diagram of the system is displayed in Figure 7. As it can be seen, the water flow was fed by a hydraulic pump working at a frequency of 40 Hz, such as measured by the flow sensor (FT01) and pressure sensor (PT01). By activating manual valves, the system can operate with one branch only or with two branches, as displayed in the piping and instrumentation diagram. Due to this configuration, it was possible to test three water demand scenarios, as discussed next.

### 4.1. Case 1: First Branch Open and Second Branch Closed

In the first case, the valve that allows the flow through the second branch was closed, so the demand to estimate was $Q_{d1}$. Then, by applying Algorithm 1, the results of the calculated pressures and flow rates are summarized in Table 2 and Table 3.

As can be seen, just by considering the pressure and flow measurement at the input, it was possible to estimate the real flow and pressure at the branch by comparing it with the readings of the pressure head meters. This scenario was the simplest and internally computed the Jacobian matrix given in (12) and the Algorithm 1.

### 4.2. Case 2: Second Branch Open and First Branch Closed

For this case, the demand to estimate was $Q_{d2}$, and the procedure was the same, with the difference that now it was $Q_{1}$ for branches one to two and $Q_{2}$ comprised the third branch. The results of the pressures and flow rates for this case are shown in Table 4 and Table 5.

### 4.3. Case 3: Both Branches Open

Finally, the valves were opened in both branches, so the flow was distributed over the entire branched network, as shown in Figure 7. The results obtained for the flows and pressures calculated in this case are given in the Table 6 and Table 7. As computed from the error rates, it is clear that the algorithm could estimate the real demand, even in the case of both branches.

**Figure 8:**
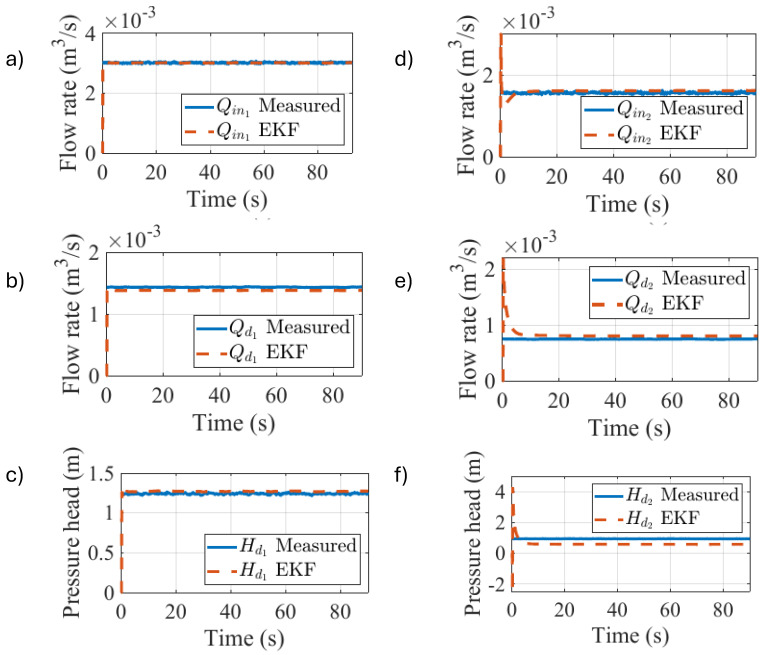
Experimental results for the EKF-driven nodal demand estimation (steady state).

However, the tables do not illustrate the convergence time of the EKF estimators, which is shown in Figure 8 for the two-branched pipeline configuration under steady-state conditions. Water was fed to the input of the pipeline ($Q_{\mathrm{in}1}$) as can be shown in plot (a) and a percentage of the total input flow deviated from the mainstream ($Q_{d_{1}}$) at the first branching, given the pressure head at the first demand node ($H_{d_{1}}$), as is visualized in plots (b,c). The remainder was considered as the input flow rate for the second demand node ($Q_{\mathrm{in}2}$), which can be seen in plot (d), where a fraction of the flow was redirected to the second branching ($Q_{d_{2}}$) given its pressure head ($H_{d_{2}}$), as is represented in plots (e) and (f), respectively.

The root of the mean squared error from the EKF-driven estimation is reported in Table 8.

**Figure 9:**
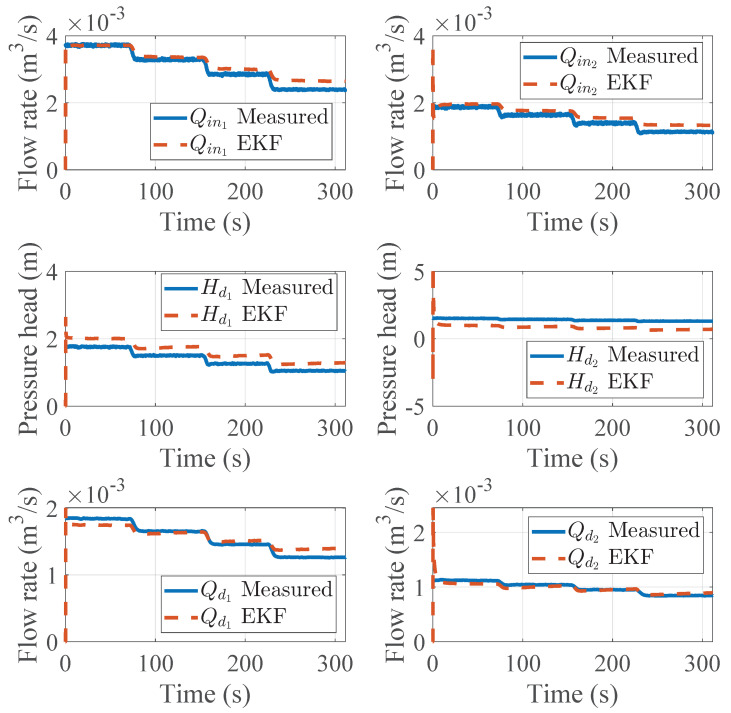
Experimental results for the EKF-driven nodal demand estimation (variable input conditions).

An additional validation test was performed using data measured at different input conditions by regulating the main pump operating point using a variable frequency drive. The EKF-driven estimation for this validation test can be seen in Figure 9.

Similarly to the first scenario, the root of the mean squared error from the EKF-driven estimation is reported in Table 9.

## 5. Conclusions

This paper presented an innovative methodology to estimate nodal demands in branched water distribution systems using an array of extended Kalman filters (EKFs). By collecting only pressure and flow measurements at the inlet point, the method accurately predicts water demands at various nodes, providing real-time data to improve system calibration and operation. Furthermore, experimental tests demonstrated that this technique was robust to noise in measurements, ensuring its reliable application under different operating conditions.

The proposed strategy is scalable due to its modular configuration, which allows estimation to be extended to multiple demand nodes. Comparing the EKF with other methods applied to similar estimation problems such as the PSO or probabilistic estimation, the EKF is computationally simpler and best suited for real-time scenarios with quick dynamics, providing a robust performance and quick convergence, relying only on pressure and flow measurements at both ends of the pipeline, which reduces the necessary instrumentation and involved costs. Future work is expected to expand the methodology to large-scale distribution networks where there is unknown demand in some nodes or sectors. This solution addresses an important gap in the existing literature, offering a practical method for online estimation, in contrast to traditional approaches that typically rely on offline simulations or models and have less applicability in real scenarios.

## Figures and Tables

**Table 1:** Physical parameter of the pilot WDS.

Parameter	Value	Units
Total length (*L*)	145.78	m
Cross-sectional area ($A_{r}$)	0.0019	m^2^
Gravity acceleration (*g*)	9.81	m/s^2^
α	100	–
$\Delta z_{1}$	28.68	m
$\Delta z_{2}$	27.22	m
$\Delta z_{3}$	28.68	m

**Table 2:** Pressure comparison case 1.

Measured Pressure Head		Estimated Pressure Head		Estimation Error
$H_{d2}$	28.8984 m	${\hat{H}}_{d2}$	29.3978 m	1.7663%
$H_{d3}$	20.1272 m	${\hat{H}}_{d3}$	20.6066 m	2.4247%

**Table 3:** Flow comparison case 1.

Measured Flow Rate		Estimated Flow Rate		Estimation Error
$Q_{1}$	0.002834 $m^{3}$/s	${\hat{Q}}_{1}$	0.002884 $m^{3}$/s	1.7725%
$Q_{2}$	0.001350 $m^{3}$/s	${\hat{Q}}_{2}$	0.001375 $m^{3}$/s	1.8989%

**Table 4:** Pressure comparison case 2.

Measured Pressure Head		Estimated Pressure Head		Estimation Error
$H_{d2}$	37.1227 m	${\hat{H}}_{d2}$	37.6023 m	1.2988%
$H_{d3}$	20.0040 m	${\hat{H}}_{d3}$	20.4594 m	2.3347%

**Table 5:** Flow comparison case 2.

Measured Flow Rate		Estimated Flow Rate		Estimation Error
$Q_{1}$	0.002532 $m^{3}$/s	${\hat{Q}}_{1}$	0.002557 $m^{3}$/s	0.9941%
$Q_{2}$	0.001331 $m^{3}$/s	${\hat{Q}}_{2}$	0.001348 $m^{3}$/s	1.3642%

**Table 6:** Pressure comparison case 3.

Measured Pressure Head		Estimated Pressure Head		Estimation Error
$H_{d2}$	23.6616 m	${\hat{H}}_{d2}$	24.1174 m	1.9263%
$H_{d3}$	15.6766 m	${\hat{H}}_{d3}$	16.00203 m	2.0758%

**Table 7:** Flow comparison case 3.

Measured Flow Rate		Estimated Flow Rate		Estimation Error
$Q_{1}$	0.002936 $m^{3}$/s	${\hat{Q}}_{1}$	0.002966 $m^{3}$/s	1.0203%
$Q_{2}$	0.001866 $m^{3}$/s	${\hat{Q}}_{2}$	0.001881 $m^{3}$/s	0.8027%
$Q_{3}$	0.000796 $m^{3}$/s	${\hat{Q}}_{3}$	0.000806 $m^{3}$/s	1.2593%

**Table 8:** Error in node-demand estimation under steady-state conditions.

Branching	RMSE
First branching ($Q_{d_{1}}$)	8.6066 × 10^−5^ m^3^/s
Second branching ($Q_{d_{2}}$)	1.3681 × 10^−4^ m^3^/s

**Table 9:** Error in nodal demand estimation under variable input conditions.

Branching	RMSE
First branching ($Q_{d_{1}}$)	9.5335 × 10^−5^ m^3^/s
Second branching ($Q_{d_{2}}$)	7.4080 × 10^−5^ m^3^/s

## Data Availability

Data are contained within the article.

## Appendix A. State Transition Function and Jacobian Matrix

From (13), defining the state vector $x={\left[Q_{\mathrm{in}},\;\lambda \left(\sigma \right)\right]}^{⊤}$ and the input $u=H_{\mathrm{in}}$, the continuous-time dynamic model for each sector of the branched pipeline can be expressed as

(A1) $$\dot{x}=\phi \left(x,u\right)=\left[\begin{array}{c} \beta \;u-\alpha \;{x_{1}}^{2}-\beta \;{x_{1}}^{2}\;x_{2}\\ 0 \end{array}\right],$$

where β is a shortcut defined as

(A2) $$\beta :=gA_{r}/\Delta z.$$

The discrete-time dynamics are obtained from the continuous-time model (A1) using Heun’s method (12). The discrete-time state transition function $\Phi :\left(x[k],u[k]\right)\to x\left[k+1\right]$ is given by

(A3) $$\Phi \left(x,u\right)=\left[\begin{array}{c} \Phi_{1}\left(x,u\right)\\ x_{2} \end{array}\right],$$

where

(A4) $$\begin{array}{cc} \Phi_{1}\left(x,u\right)= & x_{1}-{\left(\left(-\beta \;x_{2}-\alpha \right){x_{1}}^{2}+\beta \;u\right)}^{2}\left(\beta \;x_{2}+\alpha \right){T_{s}}^{3}/2\\ \; & -x_{1}\left(\left(-\beta \;x_{2}-\alpha \right){x_{1}}^{2}+\beta \;u\right)\left(\beta \;x_{2}+\alpha \right){T_{s}}^{2}\\ \; & +\left(\left(-2\;\beta \;x_{2}-2\;\alpha \right){x_{1}}^{2}+2\;\beta \;u\right)T_{s}/2 \end{array}$$

Taking the partial derivatives in (A3), the following discrete-time Jacobian matrix is obtained:

(A5) $$A=\frac{\partial \Phi (x,u)}{\partial x}=\left[\begin{array}{cc} a_{11}\left(x,u\right) & a_{12}\left(x,u\right)\\ 0 & 1 \end{array}\right],$$

where

(A6) $$\begin{array}{cc} a_{11}\left(x,u\right)= & 1+2\;x_{1}\left(\left(-\beta \;x_{2}-\alpha \right){x_{1}}^{2}+\beta \;u\right){\left(\beta \;x_{2}+\alpha \right)}^{2}{T_{s}}^{3}\\ \; & -\left(\left(-3\;\beta \;x_{2}-3\;\alpha \right){x_{1}}^{2}+\beta \;u\right)\left(\beta \;x_{2}+\alpha \right){T_{s}}^{2}-2\;x_{1}\left(\beta \;x_{2}+\alpha \right)T_{s} \end{array}$$

(A7) $$\begin{array}{cc} a_{12}\left(x,u\right)= & -\beta \;T_{s}\left(\left(\left(-\beta \;x_{2}-\alpha \right){x_{1}}^{2}+\beta \;u\right)\left(\left(-3\;\beta \;x_{2}-3\;\alpha \right){x_{1}}^{2}+\beta \;u\right){T_{s}}^{2}/2\right.\\ \; & +\left.x_{1}\left(\left(-2\;\beta \;x_{2}-2\;\alpha \right){x_{1}}^{2}+\beta \;u\right)T_{s}+{x_{1}}^{2}\right) \end{array}$$

Both the state transition function (A3) and the Jacobian matrix (A5) are evaluated in the prediction subroutine at each time step of the EKF to predict the state and the error covariance, respectively.
